# Assessment of *EGFR* Mutations in Circulating Tumor Cell Preparations from NSCLC Patients by Next Generation Sequencing: Toward a Real-Time Liquid Biopsy for Treatment

**DOI:** 10.1371/journal.pone.0103883

**Published:** 2014-08-19

**Authors:** Antonio Marchetti, Maela Del Grammastro, Lara Felicioni, Sara Malatesta, Giampaolo Filice, Irene Centi, Tommaso De Pas, Armando Santoro, Antonio Chella, Alba Ariela Brandes, Paola Venturino, Franco Cuccurullo, Lucio Crinò, Fiamma Buttitta

**Affiliations:** 1 Center of Predictive Molecular Medicine, Center of Excellence on Aging, University-Foundation, Chieti, Italy; 2 Oncological and Cardiovascular Molecular Medicine Unit, Center of Excellence on Aging, University-Foundation, Chieti, Italy; 3 Hospital “SS Annunziata”, Pathology Unit, Chieti, Italy; 4 Department of Medicine, European Institute of Oncology, Milan, Italy; 5 Department of Oncology, Humanitas Cancer Center IRCCS, Rozzano, Italy; 6 Cardiothoracic Department, University of Pisa, Pisa, Italy; 7 Department of Medical Oncology, Bellaria-Maggiore Hospital, Azienda USL of Bologna, Bologna, Italy; 8 Roche S.p.A., Monza, Italy; 9 Department of Medical Oncology, Santa Maria della Misericordia Hospital, Azienda Ospedaliera di Perugia, Perugia, Italy; Sapporo Medical University, Japan

## Abstract

**Introduction:**

Assessment of *EGFR* mutation in non-small cell lung cancer (NSCLC) patients is mandatory for optimization of pharmacologic treatment. In this respect, mutation analysis of circulating tumor cells (CTCs) may be desirable since they may provide real-time information on patient's disease status.

**Experimental Design:**

Blood samples were collected from 37 patients enrolled in the TRIGGER study, a prospective phase II multi-center trial of erlotinib treatment in advanced NSCLC patients with activating *EGFR* mutations in tumor tissue. 10 CTC preparations from breast cancer patients without *EGFR* mutations in their primary tumors and 12 blood samples from healthy subjects were analyzed as negative controls. CTC preparations, obtained by the Veridex CellSearch System, were subjected to ultra-deep next generation sequencing (NGS) on the Roche 454 GS junior platform.

**Results:**

CTCs fulfilling all Veridex criteria were present in 41% of the patients examined, ranging in number between 1 and 29. In addition to validated CTCs, potential neoplastic elements were seen in 33 cases. These included cells not fulfilling all Veridex criteria (also known as “suspicious objects”) found in 5 (13%) of 37 cases, and isolated or clustered large naked nuclei with irregular shape observed in 33 (89%) cases. *EGFR* mutations were identified by NGS in CTC preparations of 31 (84%) patients, corresponding to those present in matching tumor tissue. Twenty-five (96%) of 26 deletions at exon 19 and 6 (55%) of 11 mutations at exon 21 were detectable (P = 0.005). In 4 (13%) cases, multiple *EGFR* mutations, suggesting CTC heterogeneity, were documented. No mutations were found in control samples.

**Conclusions:**

We report for the first time that the CellSearch System coupled with NGS is a very sensitive and specific diagnostic tool for *EGFR* mutation analysis in CTC preparations with potential clinical impact.

## Introduction

The enumeration of circulating tumor cells (CTCs), rare epithelial cells identifiable in the peripheral bloodstream of cancer patients with advanced disease, has been prospectively shown to have prognostic significance for breast [1], [2], colorectal [3] and prostate cancer patients [4]. Recent data suggested a prognostic role of CTCs even in patients with non-small cell lung cancer (NSCLC) [5], [6] and small cell lung cancer [7]. The enumeration of CTCs may also allow to monitor the effectiveness of the oncological therapy in order to identify an emergent treatment resistance [3], [4], [8]. Furthermore, CTCs may be used to evaluate the expression of a number of cellular biomarkers to define the treatment with targeted therapy (Her2, BRAF) [9], [10].

The pharmacological management of patients with NSCLC is today largely based on genetic mutations that guide toward personalized therapy [11], [12]. Mutation analysis is usually performed on resected tumors, small biopsies or cytological samples from the primary neoplastic site. However, these samples could not necessarily hold genetic alterations that could later turn up during the metastatic process or be induced by pharmacological treatments [13], [14]. In addition, metastatic patients are rarely subjected to re-biopsy and even if it were to occur, a single biopsy could not represent neoplastic tissues from multiple metastatic sites because of tumor heterogeneity [15]–[17].

A further consideration is that CTCs may be also considered as a sort of “liquid biopsy” which may provide real-time information on patient's disease status [18]. In recent years, several efforts have been put into developing technologies to increase detection and characterization of CTCs from peripheral blood. The main strategies include immunomagnetic bead separation, filtration based size separation, antigens cell sorting using flow cytometry and density gradient centrifugation [19]. The CellSearch System (Veridex LLC, Raritan, NJ), based on immunomagnetic bead separation, has been approved by the U.S. Food and Drug Administration and is considered the standard method for detecting CTCs in the clinical setting. This technological platform utilizes epithelial cell-adhesion molecule (EpCAM) anti-body-coated magnetic beads to identify and enumerate CTCs. Since as few as one CTC may be found in the background of billions of peripheral white blood cells, the molecular characterization of tumor cells in blood continues to be a big challenge.

In this study, we decided to investigate the feasibility of detecting *EGFR* mutations in CTCs of NSCLC patients by coupling the CellSearch System with next generation sequencing (NGS) on the 454 GS Junior System (454 Life Sciences, Branford, CT, and Roche Applied Sciences, Indianapolis, IN). Blood samples obtained from patients enrolled in the TRIGGER study were used. TRIGGER is the acronym for an open-label, single-arm, phase II multi-center study of erlotinib (Tarceva) treatment in patients with locally advanced or metastatic (stages IIIB-IV) NSCLC who have not received previous chemotherapy for their disease and who present activating mutations in *EGFR*. The primary objective of the TRIGGER study is to evaluate the efficacy of erlotinib (Tarceva; 150 mg) on 12-month progression-free survival (PFS). Details of the study, including efficacy and safety endpoints, as well as additional secondary clinical objects, will be described in depth in a future publication. An exploratory object of the TRIGGER study was to evaluate the correlation between *EGFR* testing results obtained from basal tumor biopsies and circulating tumor cells. Here we report the results obtained by the above mentioned technological platforms on CTCs preparations from patients enrolled in the TRIGGER study.

## Materials and Methods

### Patients and blood samples

Peripheral blood samples were collected from 59 subjects including 37 NSCLC patients harboring *EGFR* mutations in primary tumor tissue, enrolled in the TRIGGER study, and 22 control cases comprising 10 breast carcinoma patients negative for *EGFR* mutations in their primary tumors and 12 healthy donors. All of the patients included in the study had locally advanced or metastatic disease (stage IIIB and stage IV) and did not receive previous chemotherapy. Peripheral blood samples were collected from patients at baseline visit in different institutions in Italy before first-line treatment with erlotinib (Tarceva). Blood samples were immediately sent at room temperature to the Center of Predictive Molecular Medicine (University-Foundation, Chieti, Italy) for CTCs counting and *EGFR* mutation analysis. Genomic DNA obtained from CTC preparations of 10 breast carcinoma patients negative for *EGFR* mutations in their primary tumors and peripheral blood buffy coat of 12 healthy donors were used as negative controls.

Written informed consent was obtained from all patients under study. Approval from independent regional Ethics Committees (Comitato Etico delle Aziende Sanitarie dell'Umbria di Perugia, Comitato per la sperimentazione clinica dei medicinali dell'Azienda Ospedaliero Universitaria pisana di Pisa, Comitato Etico della provincia di Modena, Comitato Etico dell'IRCCS Istituto Clinico Humanitas di Rozzano (MI), Comitato Etico dell'IRCCS Istituto Europeo di Oncologia di Milano, Comitato Etico della AUSL di Bologna, Comitato Etico dell'IRCCS Istituti Fisioterapici Ospitalieri di Roma) was obtained for all patients. The study was conducted in accordance with the precepts of the Helsinki Declaration.

### Whole blood collection for CTC enumeration and recovery

Blood samples (7.5 mL from each patient) were collected into CellSave blood collection tubes (Veridex LLC, Raritan, NJ). Blood samples were maintained at room temperature and processed within a maximum of 72 h after collection. Circulating tumor cells were captured by the CellSearch System (Veridex LLC, Raritan, NJ) with the CellSearch Circulating Tumor Cell Kit. CTC enumeration was performed according to Veridex criteria [20]. Other cellular elements, including suspicious cells, large naked nuclei and clustered naked nuclei, were also analyzed. Images, presented in a gallery format were independently classified by two operators according to predetermined criteria (specified by Veridex) for the presence of CTCs.

To recover CTC-enriched samples from the cartridge, an original protocol was developed. After enumeration, the supernatant was discarded maintaining the cartridge in the Magnest (Veridex LLC, Raritan, NJ) in order not to disturb the captured cells. CTC preparations were then rapidly recovered in 200 µl lysis buffer by gentle scraping of the cartridge surface with a tip, and the suspension was transferred into a tube for digestion at 55°C for 4 h. Nucleic acids were extracted from the lysed cells with the Qiagen's QIAamp DNA Micro Kit.

### PCR amplification

DNA fusions primers containing genome-specific sequences, along with one of 7 distinct 10-bp MIDs (multiplex identifier sequences used to differentiate samples being run together on the same plate) and sequencing adapters (Table S1 in File S1) were used to amplify a 108 bp region in exon 19 and a 129 bp region in exon 21 of the *EGFR* gene (NM_005228.3) as described in Methods S1 in File S1.

Different strategies were adopted to avoid cross-contaminations as previously described [21]: a) reactions were set up in positive-pressure hoods with UV sterilization systems to decontaminate reagents and equipment prior to carrying out PCRs; b) different hoods were used for PCR amplification of samples subjected to different runs; c) PCR reactions were conducted on 96-well plates, with a maximum of 4 samples loaded per plate.

### Next Generation Sequencing and analysis of sequence data

PCR products were processed before NGS as described in Supporting Information (Method S1 in File S1). A mean of 500.000 enriched beads was used for massively parallel pyrosequencing in a Titanium PicoTiterPlate (PTP) with Titanium reagents (Roche Diagnostics), on the GS Junior instrument, according to the 454 GS Junior Titanium Series Amplicon Library Preparation Method Manual (available online: www.454.com). Processed and quality-filtered reads were analysed with the GS Amplicon Variant Analyzer (AVA) software version 2.7 (454 Life Sciences). *EGFR* exons 19 and 21 reference sequences were extracted from Hg19 Human Genome Version together with both neighbor intronic regions. Such sequences were used as Reference Sequences to align every reads and the final alignments were checked manually. NGS analysis was repeated in cases with mutations in less than 1% of the DNA molecules to differentiate real mutations from low-level errors introduced during PCR amplification and sequencing. All identified mutations were searched in the online COSMIC database (http://cancer.sanger.ac.uk/cancergenome/projects/cosmic).

### Statistical analysis

The variables measured in the study were investigated for association by the Fisher's exact test or χ2 test as appropriate. A *P*<0.05 was considered as significant. Statistical analyses were performed using SPSS version 15 (SPSS, Chicago, IL).

## Results

### Preparation of CTC-enriched samples and enumeration of CTCs

**Figure 1:**
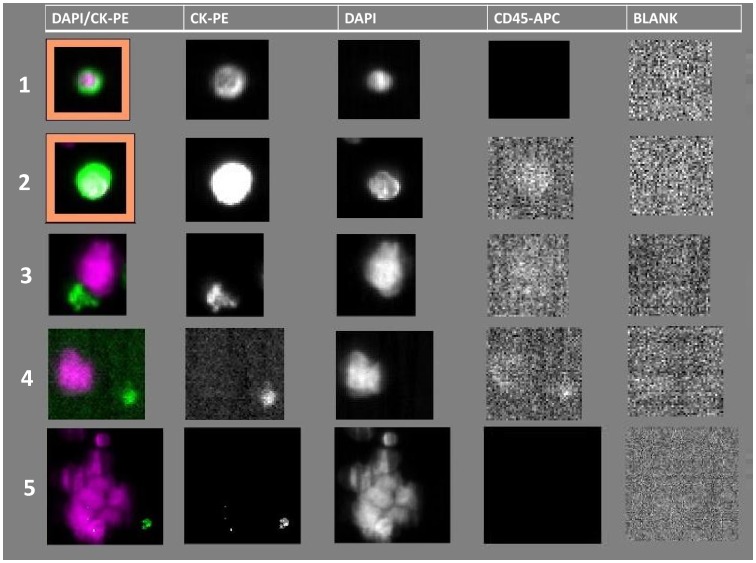
Different types of events presented by the CellSearch System. 1–2. Two classical examples of CTC fulfilling all the Veridex criteria: A) intact round to oval cells positive for epithelial cell marker (CK-PE) of more than 4 µm in size; B) positivity for the nuclear dye (DAPI) in an area smaller than the cytoplasmic area inside the cytoplasm (at least 50%); C) Negativity for the leucocyte marker (CD45/APC); D) negativity in the blank channel. 3. A suspicious object satisfying only A, C, and D criteria. 4. A large naked nucleus. 5. A cluster of naked nuclei.

Forty-nine peripheral blood samples obtained from 37 patients enrolled in the TRIGGER trial and 12 healthy donors (control cases) were examined. The median number of nuclei (DAPI+events) in the cartridge was 10,780+/−1,872. Tumor cells fulfilling all Veridex criteria were present in 15 (41%) of 37 patients examined, ranging in number between 1 and 29. In addition to validated CTCs, potential neoplastic elements were seen in 33 cases. These included cells not fulfilling all Veridex criteria (also known as “suspicious objects” according to the CellSearch training book) found in 5 (13%) of 37 cases, and isolated or clustered large naked nuclei with irregular shape observed in 33 (89%) cases (Figure 1).

### Recovery of CTC-enriched samples and evaluation of the detection sensitivity of next generation sequencing assay

An original protocol was devised to recover CTC-enriched samples from the cartridge (Veridex) and purify genomic DNA after the enumeration step as described in detail in Materials and Methods. DNA was subjected to PCR amplification and prepared for deep next generation sequencing (Roche 454 platform) analysis.

Before processing the samples from the TRIGGER study, we evaluated the detection sensitivity of NGS by dilution experiments. A genomic lung tumor DNA carrying an *EGFR* exon 19 deletion in about 50% of the molecules, as detected by NGS, was progressively diluted (1∶10, 1∶100, 1∶1.000, 1∶5.000, 1∶10.000, 1∶20.000) in wild type DNA. For each dilution experiment, 2–3 replicates were conducted as previously reported [21]. Deep sequencing was conducted performing a mean of 19,870−/+1,350 sequences per sample (about 20,000×). We were able to detect the mutation up to a dilution of 1∶10.000. Results were comparable in the different replicates, with minimal variations in the percentage of mutated molecules indicating that, at least within the range of the DNA and primers concentrations used in our study, the PCR amplification was not biased.

### *EGFR* mutation analysis by next generation sequencing

In the series of 37 NSCLC tissue samples, mutation analysis of *EGFR* was conducted in the collaborating clinical centers by Sanger sequencing or other conventional techniques and confirmed by Sanger sequencing. Of these cases, 26 (70%) were found to carry a deletion at exon 19 and 11 (30%) a mutation in exon 21.

Mutation analysis of the 37 CTC-enriched specimens by NGS was conducted blindly in the Center of Predictive Molecular Medicine of Chieti, performing a mean of 10,188−/+641 sequences per sample. *EGFR* mutations were observed in 31 (84%) of the samples examined, 25 (81%) in frame deletions at exon 19 and 6 (19%) point mutations at exon 21 (Table 1). All identified mutations had been previously reported in COSMIC database (http://cancer.sanger.ac.uk/cancergenome/projects/cosmic). Twenty-five (96%) of the 26 deletions at exon 19 and 6 (55%) of the 11 mutations at exon 21 were detected by NGS in CTC preparations (*P* = 0.005). The cases found to be negative by NGS in CTC preparations were investigated with COBAS (Roche Molecular Diagnostics, Pleasanton, CA), a sensitive real-time-based technology. No mutations were detected by COBAS in these cases.

**Table 1:** Comparison of *EGFR* mutations in primary tumors and CTC preparations.

Case	Exon	*EGFR* Mutation detected in primary tumor by SS	*EGFR* Mutation in CTC preparations** by NGS	Percentage of mutation (NGS)
#1	19	p.E746_A750del	p.E746_A750del	1.4%
#2	21	p.L858R	p.L858R	0.64%
#3	19	p.E746_A750del	p.E746_A750del	19.95%
#4	21	p.L858R	p.L858R	0.47%
#5	19	Exon 19 deletion N.O.S.	p.E746_A750del	8.45%
#6	19	p.E746_A750del	p.E746_A750del	2.35%
#7	19	Exon 19 deletion N.O.S.	p.E746_A750del	0.59%
#8	19	p.E746_S752del	p.L747_A750del>P	5.81%
#9	19	p.E746_A750del	p.E746_A750del	23.88%
#10	19	p.E746_A750del	p.E746_A750del	0.73%
#11*	21	p.A871G	p.A871G, p.L858R	0.30%
			p.A871G	0.08%
			p.L858R	0.05%
#12	21	p.L858R	WILD TYPE	/
#13	19	p.E746_A750del	p.E746_A750del	1.5%
#14	19	p.E746_A750del	p.E746_A750del	13.63%
#15	19	p.E746_A750del	p.E746_A750del	7.51%
#16*	21	p.L858R	p.L858R	2.98%
			p.L861Q	5.77%
#17	19	p.E746_A750del	WILD TYPE	/
#18	19	p.E746_A750del	p.E746_A750del	0.73%
#19	21	p.L858R	L858R	0.03%
#20	21	p.L858R	WILD TYPE	/
#21	19	p.E746_A750del	p.E746_A750del	19.3%
#22	19	p.E746_A750del	p.E746_A750del	1.43%
#23	19	p.L747_P753del>S	p.L747_P753del>S	1.67%
#24	19	p.E746_A750del	p.E746_A750del	0.61%
#25*	19	p.E746_A750del	p.E746_A750del	22.93%
			p.L747_S752del	4.05%
			p.L747_T751del	2.78%
#26	21	p.L858R	WILD TYPE	/
#27	19	p.L747_P753del>S	p.L747_P753del>S	0.75%
#28	19	p.E746_A750del	p.E746_A750del	0.71%
#29	19	p.E746_A750del	p.E746_A750del	6.35%
#30	19	p.E746_A750del	p.E746_A750del	3.44%
#31*	19	p.E746_A750del	p.E746_A750del	17.34%
			p.L747_S752del	9.03%
			p.L747_P753del>S	3.51%
#32	21	p.L861Q	WILD TYPE	/
#33	19	p.L747_A750del	p.E746_A750del	1.97%
#34	21	p.L858R	p.L858R	0.02%
#35	19	p.E746_A750del	p.E746_A750del	1.5%
#36	19	p.E746_A750del	p.E746_A750del	24.79%
#37	21	p.L858R	WILD TYPE	/

Footnotes: CTCs, Circulating Tumor Cells; SS, Sanger sequencing; NGS, next generation sequencing.

*case with double or multiple mutations.

**blood-derived material in the Cell Search cartridge containing CTCs or potential neoplastic elements.

The percentage of *EGFR* mutated molecules in CTC-enriched samples ranged between 0.02% and 24.79% with a mean of 6.34%. In all cases with mutations in less than 1% of the DNA molecules reported in Table 1, the analysis was repeated to differentiate somatic mutations from low-level errors introduced during NGS errors and the mutation was confirmed.

In 29 (94%) of the 31 cases the mutation type detected by NGS on CTCs corresponded to that found in matching tumor tissue by Sanger sequencing. In two cases (#8 and #33, Table 1), carrying an *EGFR* deletion at exon 19, the deletion detected by NGS was similar, but not exactly corresponding to that observed by Sanger sequencing. Of the two mutations identified by Sanger sequencing one has never been reported before in Cosmic Database, the other was reported as an extremely rare mutation type. These two cases could represent reading errors of Sanger sequencing as we have previously shown in a dedicated study [21]. The NGS was repeated in these two cases and the type of mutation confirmed.

**Figure 2:**
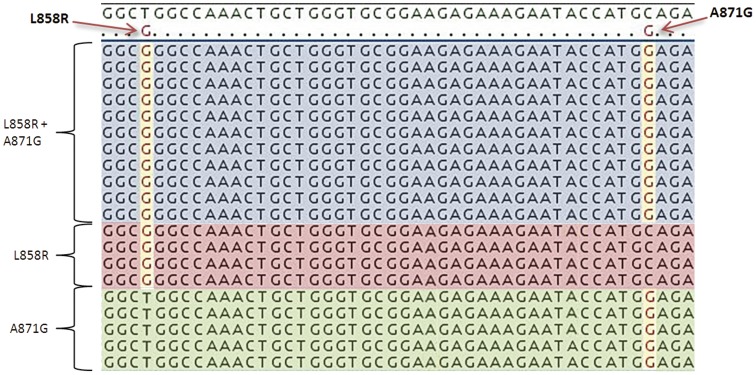
EGFR Mutation Heterogeneity in Circulating Tumor Cells. Sequences of the 3 different alleles, observed in case #11 by next generation sequencing, suggesting a genetic heterogeneity for *EGFR* mutations in CTCs (see text). The 3 alleles are shown in different colors.

In 4 (13%) cases (#11, #16, #25 and #31) carrying *EGFR* alterations in CTCs, double or multiple mutations were observed by NGS (Table 1). In one of these cases (#11) showing a rare type of point mutation (A871G) in tumor tissue, three different alleles were observed in CTCs, one carrying the A871G mutation, one carrying the more common L858R mutation and one carrying both A871G and L858R mutations (Figure 2). This intriguing result prompted us to repeat the analysis in different blood specimens obtained from the same patient, including a plasma sample. Results were confirmed in all specimens.

No previously reported *EGFR* mutations were detected in the DNA extracted from buffy coats of the 12 peripheral blood control samples.

## Discussion

The present study was devised to evaluate the correlation between *EGFR* mutation status in basal tumor biopsies and matching circulating tumor cells of NSCLC patients. A series of peripheral blood specimens from 37 NSCLC patients, enrolled in the TRIGGER trial, carrying *EGFR* mutations in their primary tumor, were subjected to CTC preparation by the Veridex CellSearch System and investigated for *EGFR* mutations by next generation sequencing on the 454 GS Junior platform. DNA Samples from CTC preparations of 10 breast carcinoma patients negative for *EGFR* mutations in their primary tumors and buffy coats from 12 healthy subjects, were also investigated as negative controls.

The new technical approach utilized, based on pyrosequencing of emulsion PCR reactions, is one of the most sensitive methods available for the detection of somatic mutations when used in ultra-deep sequencing [21]–[23]. In addition, NGS has several advantages over other sensitive mutation detection techniques including: a) it is a screening technique allowing to detect all type of mutations in a given PCR amplified DNA fragment; b) it can allow to get information on genetic heterogeneity in CTC preparations. We decided to perform an ultra-deep NGS analysis taking a median of more than 10.000 sequences per sample. Ultra-deep NGS allowed the detection of *EGFR* mutations in 84% of the CTC samples examined, while no mutations were seen in the series of control samples. Our data indicate that the CellSearch System coupled with ultra-deep sequencing represents a powerful method for the detection of *EGFR* mutations in CTCs with sensitivity and specificity of 84% and 100%, respectively. At the moment NGS has a number of drawbacks that limit its application for routine monitoring of patient's disease status in that it is labor intensive and relatively expensive. However, other reliable and easy-to-use tests, although less sensitive in our hands, can be utilized for routine monitoring in clinical practice. The possibility to detect genetic mutations in CTCs may have several clinical advantages over conventional mutation detection in tissue: 1) blood samples can be obtained easily and repeatedly, while tissue samples require invasive procedures and re-biopsies are sometimes challenging; 2) CTCs may represent the current status of the neoplastic growth and as so they could be important in monitoring for recurrences and development of drug resistance, while tissues are usually collected months or years before treatment; 3) CTCs may represent the whole neoplastic process (primary tumor/s and metastases), whereas a biopsy on a single site could not reflect the status of multiple sites.

CTC preparations, obtained by the FDA approved CellSearch System (Veridex), have been used as a source of nucleic acids for mutation analysis in only two previous studies. Jiang *et al.* reported the possibility to detect *Androgen Receptor* (*AR*) mutations in CTCs prepared by the Profile Kit (Veridex) in a series of 35 castration-resistant prostate cancer patients [18]. By using the Transgenomic's WAVE denaturing HPLC technology followed by direct sequencing, capable of detecting mutant species at relative abundances as low as 2.5%, *AR* mutations were detected in 57% of the patients. More recently, Punnoose *et al.* evaluated the possibility to detect *EGFR* mutations in CTCs of NSCLC patients using the same approach for CTC preparation and a real-time quantitative TaqMan assay [24]. Among 8 cases carrying *EGFR* mutations in primary tumors only one case was found to be positive for *EGFR* mutation in CTCs. The authors concluded that mutational analysis of CTCs captured on the CellSearch platform was challenging because of the mutation assays used in their study which had a sensitivity of 1% to 5% in a background of wild-type DNA [15]. It is possible that wild-type copies of the gene of interest from contaminating white blood cells co-isolated with CTCs using the CellSearch System might have obscured the signal from CTCs. In our study, we devised a method to prepare CTCs for DNA extraction directly from the cartridge included in the enumeration kit. This approach allowed us to evaluate the number of all presented events and the morphology of the cells before DNA extraction. In addition, we used, for the first time on enriched CTCs, an extremely sensitive ultra-deep NGS assay. CTC preparations contained a large excess of normal white blood cells, with a median number of 11.000 DAPI positive events. CTCs validated according to Veridex criteria or potential neoplastic elements (suspicious objects and large naked nuclei) were present in all of the samples analyzed. The ratio between validated CTCs and white blood cells was under 1∶100 in all cases investigated. However, the high sensitivity of ultra-deep NGS allowed the detection of *EGFR* mutations in the vast majority of the samples, albeit at or below 1%. This low percentage of mutant allele could be ascribed to normal blood cells captured in the CellSearch cartridge non-specifically contributing DNA to the sample. *EGFR* mutations were observed in 13 (87%) of 15 cases positive for CTCs fulfilling all Veridex criteria, and in 16 (73%) of 22 cases showing only potential neoplastic elements. A similar finding has previously been reported by Jiang *et al.* [18], who found *AR* mutations in cases that were found to be negative for CTCs. According to the results obtained in our series, we speculate that potential neoplastic elements in the cartridge may represent the source of DNA carrying *EGFR* mutations in samples lacking CTCs validated by Veridex criteria.

Working on CTC nowadays is not easy, time-consuming and expensive. Plasma samples may represent at the moment a valid alternative. However, the use of new generation platforms for CTC isolation could further enhance the possibility of detecting mutations in CTCs. Promising results have been reported by means of a microfluidic-based device (CTC-chip) that can isolate, quantify, and analyze circulating tumor cells from blood sample [25]–[28]. Although potentially useful, this new device is not commercially available at the present time.

A high degree of concordance between mutation data in tumor tissue and CTC preparations was observed for *EGFR* exon 19 deletions. The concordance in case of other types of mutations was significantly lower. These differences could be ascribed to possible technical shortcoming of the 454 NGS technology. To further address this point, we decided to investigate the cases found to be negative by NGS in CTC preparations with COBAS, a sensitive real-time-based technology, successfully used for the detection of *EGFR* mutations in plasma samples by other groups [29]. No mutations were detected by COBAS in these samples. On the basis of our findings, we are tempted to hypothesize that the increased copy number of *EGFR*, more frequently associated with deletions at exon 19 [30], could facilitate their detection in CTCs.

In 4 cases (13%) double or multiple *EGFR* mutations were found by NGS in CTCs obtained from patients carrying a single *EGFR* mutation, detected by Sanger sequencing, in the corresponding tumor biopsy. Particularly interesting was the case of a patient (case #11, Table 1) with a rare type of point mutation (A871G) in the tumor sample. In the CTC-enriched sample of this patient, 3 different types of alleles were found, one affected by the A871G mutation, one carrying the more common L858R mutation and one showing both A871G and L858R mutations (Figure 2). Our result strongly suggests a genetic heterogeneity for *EGFR* mutations in CTCs. Multiple *EGFR* mutations in lung cancer have previously been observed in tumors tissues by ultra deep sequencing [21] as well as in circulating tumor cells [26], [31]. The presence of genetic heterogeneity in CTCs is in keeping with the observation that the mutational status of *EGFR* is sometimes different in the primary lesion and metastatic sites. Tumor heterogeneity may be implicated in pharmacological resistance. In particular, it has been reported that the coexistence of the sensitive classical mutation L858R with mutations at codon 871 is associated with disease progression after treatment with TKIs [32], [33]. In these 4 cases, however, we can not exclude the possibility that multiple mutations observed by NGS in CTC preparations could have been present in tumor tissue in minor clones not detectable by Sanger Sequencing.

In conclusion, we report for the first time that CTC preparations obtained by the CellSearch platform represent a suitable source of tumor DNA for an efficient detection of *EGFR* mutations by ultra-deep next generation sequencing. The innovative diagnostic approach described could be particularly useful in cases with very limited amount of biological material or to monitor the mutational status of the tumor during treatment, with special emphasis on the presence of mutations involved in acquired resistance to TKIs.

## Supporting Information

File S1 **PCR amplification, Next Generation Sequencing, and analysis of sequence data.**
(DOC)
