# Supplementary material for: Assessment of EGFR Mutations in Circulating Tumor Cell Preparations from NSCLC Patients by Next Generation Sequencing: Toward a Real-Time Liquid Biopsy for Treatment
Source: PLoS One. 2014 Aug 19;9(8):e103883. doi: 10.1371/journal.pone.0103883 (PMC4138040; doi:10.1371/journal.pone.0103883)
Supplement: File S1 — PCR amplification, Next Generation Sequencing, and analysis of sequence data. (DOC) [file pone.0103883.s001.doc]

**Combined Supporting Information (File S1)**

Methods S1

***PCR amplification, Next Generation Sequencing, and analysis of sequence data***

After DNA extraction and quantification, the CTC-enriched samples were subjected to PCR amplification and deep sequencing by the 454 GS Junior System (454 Life Sciences, Branford, CT, and Roche Applied Sciences, Indianapolis, IN). The 454 GS Junior technology is derived from the technological convergence of emulsion PCR and pyrosequencing.

Briefly, PCR reactions were performed by fusions primers containing genome-specific sequences, along with one of 7 distinct 10-bp MIDs (multiplex identifier sequences used to differentiate samples being run together on the same plate) and sequencing adapters (see Supplemental Table S1), specially devised for *EGFR* exon 19 and 21. PCR primers, were designed using the OligoAnalyzer 3.1 software (http://eu.idtdna.com/analyzer/Applications/OligoAnalyzer/) and synthesized at MWG-Biotech AG.

The sequence amplicons were of 108 bp for exon 19 and 129 bp for exon 21. PCR reactions were run in 30 μl reaction volumes, containing 5.5 mmols dNTPs, 11 μmols of each primer, 2.75 μl PCR buffer, 1 μl DNA, and 1.3 units of FastStart HiFi Polymerase (Roche Diagnostics). A touch-down PCR cycling program was performed on the Gene Amp PCR system 9700 thermocycler (Applied Biosystems) with an initial step at 94°C for 2 min followed by 43 cycles at 94°C for 30 sec, 64°C (decreasing the temperature by 1°C each cycle for six cycles) for 30 sec, and 70°C for 30 sec, and a final step at 70°C for 5 min.

A number of strategies were adopted to avoid cross-contaminations among different samples and different runs: a) reactions were set up in positive-pressure hoods with UV sterilization systems to decontaminate reagents and equipment prior to carrying out PCRs; b) different hoods were used for PCR amplification of samples subjected to different runs; c) PCR reactions were conducted on 96-well plates, with a maximum of 4 samples loaded per plate. In addition, the presence of patient-specific MID sequences within the primers allows the detection of possible cross-contamination products which are automatically recognized by the software and excluded from analysis.

PCR products were visualized on agarose gel, purified using Ampure-XP DNA-binding paramagnetic beads (Agencourt Bioscience Corp., Beckman Coulter S.p.A, Milan, Italy), and quantified in 96-well format with the QuantiFluor™-ST Fluorometer (Promega, Madison, Wisconsin, USA) using a PicoGreen® assay (Invitrogen, Carlsbad, CA). Samples were then diluted to an approximate concentration of 1 × 10^9^ molecules/μL and pooled at equimolar concentrations to create a highly multiplexed amplicon library. After pooling, the library was further diluted to 10^6^ molecules/µl and subjected to emulsion PCR (emPCR) using the 454 GS Junior Titanium Series Lib-A emPCR Kit (Roche Diagnostics), according to the manufacturer’s protocols. Following emPCR, the captured beads with bound DNA were enriched with a second DNA capture mechanism to separate out beads with and without bound emPCR products.

A mean of 500.000 enriched beads was used for massively parallel pyrosequencing in a Titanium PicoTiterPlate® (PTP) with Titanium reagents (Roche Diagnostics), on the GS Junior instrument, according to the 454 GS Junior Titanium Series Amplicon Library Preparation Method Manual (available online: www.454.com). Processed and quality-filtered reads were analysed with the GS Amplicon Variant Analyzer (AVA) software version 2.7 (454 Life Sciences). *EGFR* exons 19 and 21 reference sequences were extracted from Hg19 Human Genome Version together with both neighbor intronic regions. Such sequences were used as Reference Sequences to align every reads and the final alignments were checked manually. NGS analysis was repeated in cases with mutations in less than 1% of the DNA molecules to differentiate real mutations from low-level errors introduced during PCR ampliﬁcation and sequencing.

**Table S1: List of forward and reverse fusion primers utilized for PCR amplification of *EGFR*  exon 19 and 21.**

| **Fusion Primers Ex19 *EGFR*** | | |
| --- | --- | --- |
| **MID 1** | FW  Rev | 5’-CGTATCGCCTCCCTCGCGCCATCAG *ACGAGTGCGT* **CCCAGAAGGTGAGAAAGTTAAAATTC**-3’  5’-CTATGCGCCTTGCCAGCCCGCTCAG *ACGAGTGCGT* **ACAGCAAAGCAGAAACTCACAT**-3’ |
| **MID 2** | FW  Rev | 5’-CGTATCGCCTCCCTCGCGCCATCAG *ACGCTCGACA* **CCCAGAAGGTGAGAAAGATAAAATTC**-3’  5’-CTATGCGCCTTGCCAGCCCGCTCAG *ACGCTCGACA* **ACAGCAAAGCAGAAACTCACAT**-3’ |
| **MID 3** | FW  Rev | 5’-CGTATCGCCTCCCTCGCGCCATCAG *AGACGCACTC* **CCCAGAAGGTGAGAAAGATAAAATTC**-3’  5’-CTATGCGCCTTGCCAGCCCGCTCAG *AGACGCACTC* **ACAGCAAAGCAGAAACTCACAT**-3’ |
| **MID 4** | FW  Rev | 5’-CGTATCGCCTCCCTCGCGCCATCAG *AGCACTGTAG* **CCCAGAAGGTGAGAAAGATAAAATTC**-3’  5’-CTATGCGCCTTGCCAGCCCGCTCAG *AGCACTGTAG* **ACAGCAAAGCAGAAACTCACAT**-3’ |
| **MID 5** | FW  Rev | 5’-CGTATCGCCTCCCTCGCGCCATCAG *ATCAGACACG* **CCCAGAAGGTGAGAAAGATAAAATTC**-3’  5’-CTATGCGCCTTGCCAGCCCGCTCAG *ATCAGACACG* **ACAGCAAAGCAGAAACTCACAT**-3’ |
| **MID 6** | FW  Rev | 5’-CGTATCGCCTCCCTCGCGCCATCAG *ATATCGCGAG* **CCCAGAAGGTGAGAAAGATAAAATTC**-3’  5’-CTATGCGCCTTGCCAGCCCGCTCAG *ATATCGCGAG* **ACAGCAAAGCAGAAACTCACAT**-3’ |
| **MID 7** | FW  Rev | 5’-CGTATCGCCTCCCTCGCGCCATCAG *CGTGTCTCTA* **CCCAGAAGGTGAGAAAGATAAAATTC**-3’  5’-CTATGCGCCTTGCCAGCCCGCTCAG *CGTGTCTCTA* **ACAGCAAAGCAGAAACTCACAT**-3’ |
|  | | |
| **Fusion Primers Ex21 *EGFR*** | | |
| **MID 1** | FW  Rev | 5’-CGTATCGCCTCCCTCGCGCCATCAG *ACGAGTGCGT* **CCGCAGCATGTCAAGATCACAGA**-3’  5’-CTATGCGCCTTGCCAGCCCGCTCAG *ACGAGTGCGT* **CCTGGTGTCAGGAAAATGCTGG**-3’ |
| **MID 2** | FW  Rev | 5’-CGTATCGCCTCCCTCGCGCCATCAG *ACGCTCGACA* **CCGCAGCATGTCAAGATCACAGA**-3’  5’-CTATGCGCCTTGCCAGCCCGCTCAG *ACGCTCGACA* **CCTGGTGTCAGGAAAATGCTGG**-3’ |
| **MID 3** | FW  Rev | 5’-CGTATCGCCTCCCTCGCGCCATCAG *AGACGCACTC* **CCGCAGCATGTCAAGATCACAGA**-3’  5’-CTATGCGCCTTGCCAGCCCGCTCAG *AGACGCACTC* **CCTGGTGTCAGGAAAATGCTGG**-3’ |
| **MID 4** | FW  Rev | 5’-CGTATCGCCTCCCTCGCGCCATCAG *AGCACTGTAG* **CCGCAGCATGTCAAGATCACAGA**-3’  5’-CTATGCGCCTTGCCAGCCCGCTCAG *AGCACTGTAG* **CCTGGTGTCAGGAAAATGCTGG**-3’ |
| **MID 5** | FW  Rev | 5’-CGTATCGCCTCCCTCGCGCCATCAG *ATCAGACACG* **CCGCAGCATGTCAAGATCACAGA**-3’  5’-CTATGCGCCTTGCCAGCCCGCTCAG *ATCAGACACG* **CCTGGTGTCAGGAAAATGCTGG**-3’ |
| **MID 6** | FW  Rev | 5’-CGTATCGCCTCCCTCGCGCCATCAG *ATATCGCGAG* **CCGCAGCATGTCAAGATCACAGA**-3’  5’-CTATGCGCCTTGCCAGCCCGCTCAG *ATATCGCGAG* **CCTGGTGTCAGGAAAATGCTGG**-3’ |
| **MID 7** | FW  Rev | 5’-CGTATCGCCTCCCTCGCGCCATCAG *CGTGTCTCTA* **CCGCAGCATGTCAAGATCACAGA**-3’  5’-CTATGCGCCTTGCCAGCCCGCTCAG *CGTGTCTCTA* **CCTGGTGTCAGGAAAATGCTGG**-3’ |

**Note**: Starting at the 3’-end of each primer: a 22-26 bp target-specific segment, a 10 bp sequence (MID) specific for each patient in each run and a common 25 bp region used in clonal amplification and sequencing reactions at the 5′-end.

**Abbreviations**: MID, Multiplex Identifier; FW, forward; Rev, reverse.
